# Joubert’s syndrome and related disorders and home-based peritoneal dialysis in East Africa: a case report

**DOI:** 10.1186/s13104-017-3033-7

**Published:** 2017-12-06

**Authors:** Grace M. Musiime, Doris M. W. Kinuthia, Donald P. Oyatsi, Wangui Manguyu

**Affiliations:** 1 Stellenbosch University Faculty of Medicine and Health Sciences, Francie van Zijl Drive, Tygerberg, Cape Town, 7505 South Africa; 2 Gertrude’s Children’s Hospital, Muthaiga Road, P.O Box 42325, Nairobi, 00100 Kenya

**Keywords:** Joubert’s syndrome, Peritoneal dialysis, Sub-Saharan Africa, Case report

## Abstract

**Background:**

Joubert’s syndrome is a rare condition affecting an estimated 1:80,000–1:100,000 individuals. There is underdevelopment of the cerebellar vermis resulting in a characteristic molar tooth sign on cross sectional axial magnetic resonance imaging. It can occur in association with multi-organ involvement; in such cases it is classified as Joubert’s syndrome and related disorders. To date, there are no cases of Joubert’s syndrome and related disorders from sub-Saharan Africa described in the literature.

**Case presentation:**

An 8 year old black Kenyan female child was diagnosed in Joubert’s syndrome in her first year of life. She was noted to have dysmorphic facies and hypotonia in the neonatal period and cranial MRI showed dysplasia of the cerebellar vermis and typical molar tooth malformation. She was subsequently lost to follow up for several years and later presented with anaemia. Further investigation revealed bilateral multicystic kidneys and significant renal impairment consistent with a diagnosis of end stage renal failure and polycystic kidney disease. She underwent home peritoneal dialysis for 7 months.

**Conclusions:**

Joubert’s syndrome and related disorders is a rare condition. This case report demonstrates that home peritoneal dialysis is feasible in a low resource setting. Although it is scarcely provided in African countries, it is an effective renal replacement strategy for patients with end stage renal disease.

## Background

Joubert’s syndrome (JS) is a rare condition affecting an estimated 1/100,000–1/250,000 individuals; this is likely an underestimate as the exact prevalence remains unknown [1]. Inheritance is predominantly autosomal recessive although a few X-linked recessive cases have been reported [1–4]. JS can occur in association with multiorgan involvement. In such cases it is defined as Joubert’s syndrome and related disorders (JSRD) [2–4]. There are six phenotypes of JSRD [2]. The characteristic molar tooth sign of elongated, thickened superior cerebellar peduncles, vermal hypoplasia or aplasia and deepening of the interpeduncular fossa is seen on computed tomography (CT) or magnetic resonance imaging (MRI) of the brain in all cases [1, 3, 5].

Chronic kidney disease (CKD) is a significant and potentially increasing public health burden in sub-Saharan Africa (SSA) [6, 7]. A recent systematic review estimates the prevalence of CKD in SSA is approximately 13.8%, although there is significant inter-country variation [6]. Treatment of end stage renal disease is challenging, due to large patient numbers and limited facilities, financial support and expertise; less than 2% of patients needing dialysis in Africa are able to access treatment although availability of services varies across countries [7–9]. A majority of patients undergo in-centre haemodialysis (HD) [10].

We describe a case of JSRD and home peritoneal dialysis (PD) in Kenya. To the best of our knowledge, this is the first documented case of JSRD and home-based peritoneal dialysis in a child in East Africa. Additionally, we were unable to locate any case reports of JSRD from SSA in the published literature.

## Case presentation

An 8 year old black Kenyan female child was diagnosed with JS in her first year of life. She was delivered at term via spontaneous vaginal delivery with a birth weight of 3.2 kg. There was no perinatal resuscitation and antenatal history was unremarkable. On her second day of life, she had multiple generalised tonic–clonic convulsions. The patient had 16 year old brother who is presently alive and well. Two siblings died as infants; the first died at 3 weeks of age and had an encephalocoele. The other sibling died at 9 months of age and had an encephalocoele and hydrocephalus for which a ventriculoperitoneal shunt was inserted.

**Fig. 1:**
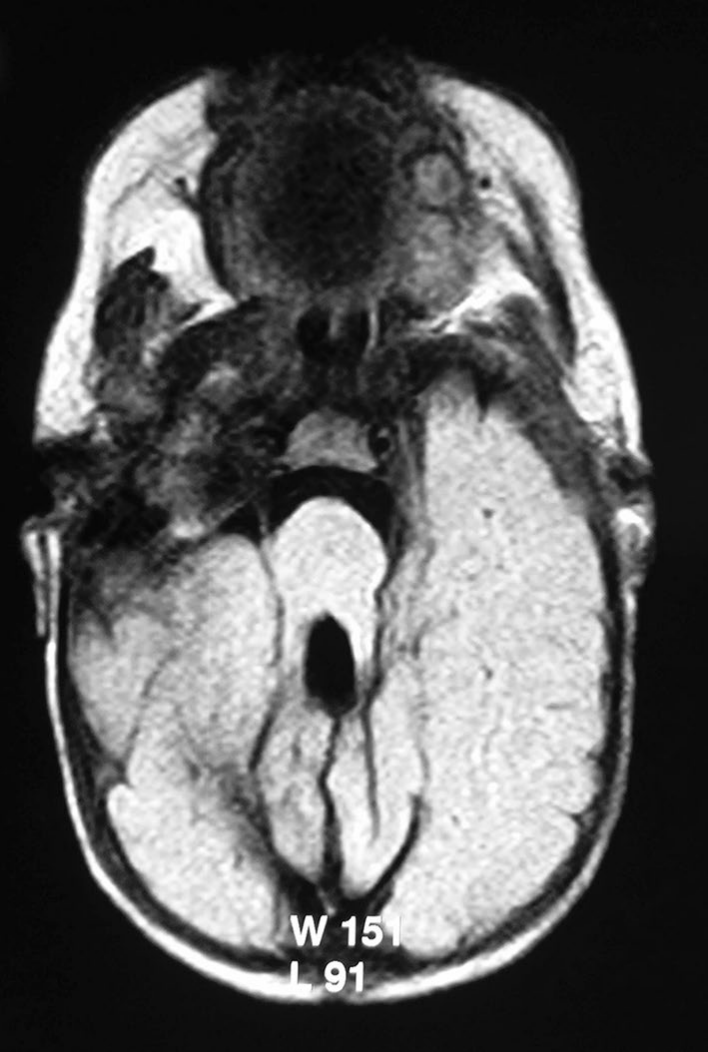
MRI brain, age 56 days. Axial section showing small dysplastic vermis, large rounded superior cerebellar peduncles and absence of decussation of white matter pathways forming the ‘molar tooth’ malformation

On examination she was noted to have generalised hypotonia with a flat nasal bridge, abnormal eye movements and both anterior and posterior fontanelles were enlarged. MRI brain done at 56 days of age showed a small dysplastic vermis, large rounded superior cerebellar peduncles and absence of decussation of white matter pathways forming the ‘molar tooth’ malformation, characteristic of JS (Fig. 1). The fourth ventricle was enlarged; there was ventricular asymmetry and dilatation with absence of septum pellucidum and a midline lipoma in the occipital region. Oral phenobarbital and phenytoin were commenced after which no further convulsions were noted. She was followed up by a paediatric neurologist until 2 years of age at which point she was lost to follow up.

She presented several years later, age 7 years, for a general outpatient review. She was not on any medication and had reportedly been seizure free for several years. She had characteristic clinical features of JS, namely; gross developmental delay and cognitive impairment, hypotonia with abnormal limb movements and generalised muscle wasting, a wide nasal bridge, low set ears, a protruding tongue and abnormal eye movements. Her weight at this point was 17 kg and length 119 cm. A macrocytic anaemia was noted on full blood count (haemoglobin 6.2 g/dL, reference range 11.5–15.5 g/dL; mean corpuscular volume 99 fL, reference range 78–94 fL). Further investigation revealed hypothyroidism (thyroid stimulating hormone 2.53 U/mL, reference range 0.6–5.1 U/mL; free triiodothyronine 2.22 pg/mL, reference range 2.6–5.4 pg/mL; free thyroxine 0.69 ng/dL, reference range 0.8–2.05 ng/dL). Peripheral blood film showed macrocytic normochromic red blood cells of normal morphology and haematinics were within normal limits. She was transfused with packed red blood cells and started on levothyroxine 25 μg once daily.

**Fig. 2:**
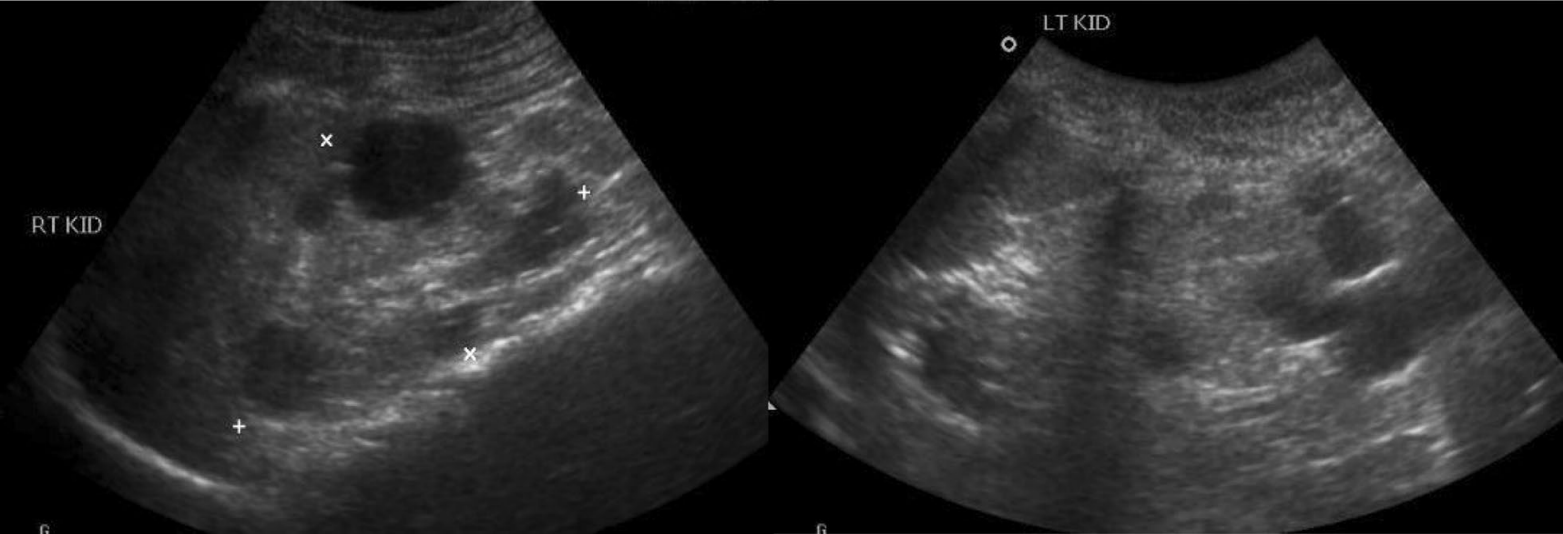
Renal ultrasound, age 7 years. Both kidneys are 9 × 3.5 cm. Multiple cortical cysts of variable size are seen in both kidneys

One month later she presented to our institution following two generalised tonic–clonic convulsions. No acute changes were noted on CT brain. She was started on oral phenobarbital 30 mg once daily and carbamazepine 100 mg once daily. Her haemoglobin was 6.4 g/dL and marked renal impairment was noted with urea 16.7 mmol/L (reference range 1.7–8.3 mmol/L), creatinine 460 µmol/L (reference range 27–62 µmol/L) and estimated glomerular filtration rate (eGFR) 10 mL/min/1.73 m^2^. Serum potassium was 6 mmol/L (reference range 3.5–5 mmol/L), other electrolytes were within normal limits. Renal ultrasound showed hyperechoic kidneys of size 9 × 3.5 cm with loss of normal corticomedullary differentiation, multiple cortical cysts of variable sizes in both kidneys consistent with renal parenchymal disease and bilateral polycystic kidney disease (Fig. 2). There were no peripheral stigmata of chronic renal disease noted on examination. She was transfused with packed red blood cells and started on subcutaneous erythropoietin 2000 units once weekly.

Her parents were counselled regarding the short and long term implications of her diagnosis and guarded prognosis. Following a multi-disciplinary ethical committee meeting, it was decided that dialysis should be started. Palliative care was deemed inappropriate at this stage as the patient was still at her baseline level of functioning. Her parents request for maximal intervention was also considered during the decision making process. Peritoneal dialysis was initiated in hospital and continued at home. She was deemed an unsuitable candidate for haemodialysis as she would have required sedation for each session due to frequent and erratic limb movements.

### Outcome and follow-up

She underwent home PD for a 7 month period which was manually administered by renal nurses. Her mother declined to be trained in PD administration and chose not to engage with the counselling services offered to her. During this period she had 2 week-long hospital admissions with peritonitis (culture negative) which was treated with intra-peritoneal vancomycin and intravenous ceftazidime. Her nutritional needs were met by nasogastric feeding. After 7 months of home PD, she was admitted to the paediatric intensive care unit with Stevens–Johnson syndrome. She subsequently developed severe sepsis with multiple organ failure, secondary to hospital acquired multi-drug resistant klebsiella pneumoniae. Her PD catheter was changed as it was obstructed. She died 22 days following admission in June 2016, aged 8 years and 14 days.

**Fig. 3:**
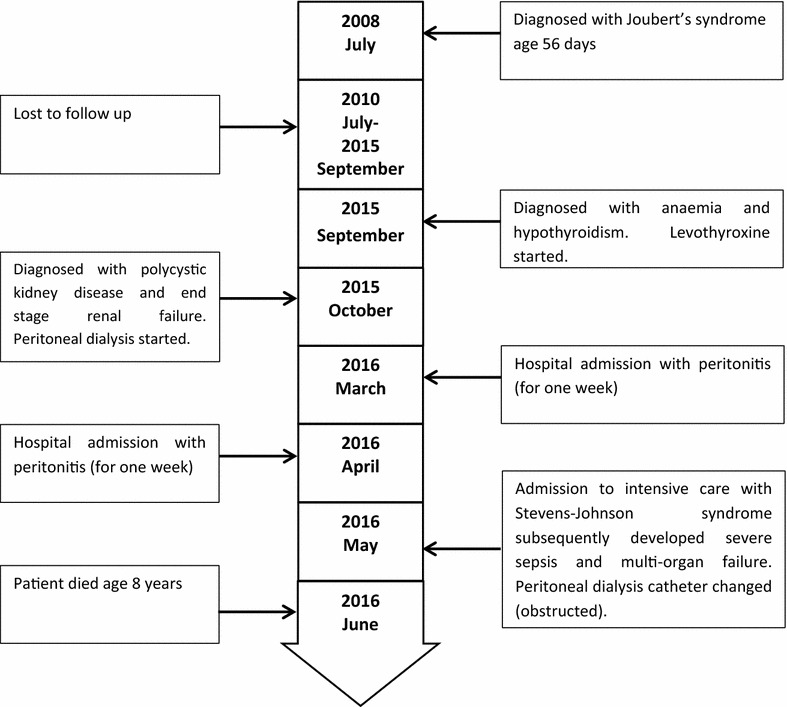
Timeline showing important clinical events during management of patient

The timeline of the case is shown in Fig. 3.

## Discussion and conclusions

Classical JS presents with hypotonia, developmental delay of varying severity, abnormal eye movements and an irregular respiratory pattern in the infantile period (intermittent tachypnoea and/or apnoea). No stringent diagnostic criteria have been established and oculomotor or respiratory abnormalities may not always be present [3]. There are six phenotypes of JSRD [2]. Ophthalmologic, renal, hepatic, musculoskeletal and endocrine anomalies can occur in JSRD [2, 4]. Renal disease is common and can occur as cystic dysplasia, polycystic kidney disease or juvenile nephronophthisis [1, 4, 11]. Endocrine abnormalities in JSRD can present as panhypopituitarism or isolated insufficiency of specific hormones, such as thyroid or growth hormone deficiency [4]. This patient had characteristic clinical features of JSRD with polycystic kidney disease and thyroid hormone deficiency.

Peritoneal dialysis is an effective but globally under utilised strategy of renal replacement therapy. There are few medical contraindications for PD and overall survival is similar to HD. It may also be possible to provide PD in more remote locations than HD, although the cost of PD is greater than that of HD in most African countries [9, 12]. Peritoneal-dialysis associated peritonitis can cause significant morbidity and has an impact on patient mortality; some episodes may require temporary or permanent transfer to HD, depending on the duration and severity of the peritonitis [13].

We have demonstrated that home PD can be successfully carried out in a child with a disability in a low resource setting. Although the decision to initiate dialysis in this case posed an ethical dilemma given the patient’s underlying diagnosis of JSRD and associated disability, home PD enabled her parents to adapt their schedule such that they were able to care for her and continue with work and other day-to-day activities. Despite stringent infection control measures and PD being administered solely by renal nurses, she had two episodes of peritonitis in a 7 months period; this infection rate is higher than that observed in Asian and Western countries [14]. We were unable to locate any data on PD associated peritonitis rates from SSA.

### Challenges of home PD in a low resource setting

Automated peritoneal dialysis machines are not readily available in Kenya or other East African countries. Thus the PD has to be manually administered, either by the primary caregiver or trained personnel. In cases such as this where caregivers decline to participate, they incur extra costs for home nursing services. Additionally the family covers the cost of the PD fluid, either directly or through private health insurance, as this is not incorporated into the state funded health insurance scheme. Disruptions to the supply chain occasionally occur; during these periods the PD fluid is difficult to source.

### Limitations of current practice

Although the absence of a structured primary healthcare system offers patients the flexibility of selecting their primary and specialist healthcare provider and health facility, continuity of care for chronic conditions is difficult to coordinate. As seen in this case prior to presenting to our institution the patient was reviewed by multiple clinicians on an outpatient basis. It is possible the renal dysfunction may have been detected sooner if the outpatient care had been coordinated by a single clinician or if an integrated case notes system for different healthcare providers was established. As facilities and specialist care services for genetic counselling and testing are not available in East Africa, the inability to provide the family with genetic counselling should be noted. Additionally, we acknowledge that mutational analysis and estimates of other pituitary hormones should ideally have been undertaken, however this was not done due to the unavailability of these tests in Kenya at the time this patient was under our care.

### Programmatic implications and recommendations for further research

In order to increase home PD coverage and quality while ensuring sustainability in low resource settings, mechanisms to minimise costs such as locally manufacturing PD fluid are needed. Training programmes for both nurses and doctors are also essential and supervision systems should be developed. If PD coverage is increased in low and middle income countries, implementation research with a particular focus on incidence and causative organisms of bacterial peritonitis should be undertaken to inform clinical practice and local guidelines.

### Conclusions

JSRD is a rare condition. Home paediatric PD, despite its challenges, is feasible in a low resource setting. It should be scaled up, where possible, to improve much needed access to renal replacement therapy.

## Abbreviations

CKD: chronic kidney disease
CT: computed tomography
HD: haemodialysis
JS: Joubert’s syndrome
JSRD: Joubert’s syndrome and related disorders
MRI: magnetic resonance imaging
PD: peritoneal dialysis
SSA: sub-Saharan Africa
